# Dysregulated Neurovascular Control Underlies Declining Microvascular Functionality in People With Non-alcoholic Fatty Liver Disease (NAFLD) at Risk of Liver Fibrosis

**DOI:** 10.3389/fphys.2020.00551

**Published:** 2020-06-03

**Authors:** Geraldine F. Clough, Andrew J. Chipperfield, Marjola Thanaj, Eleonora Scorletti, Philip C. Calder, Christopher D. Byrne

**Affiliations:** ^1^Human Development and Health, Faculty of Medicine, University of Southampton, Southampton, United Kingdom; ^2^Faculty of Engineering and Physical Sciences, University of Southampton, Southampton, United Kingdom; ^3^National Institute for Health Research Southampton Biomedical Research Centre, University of Southampton, University Hospital Southampton National Health Service Foundation Trust, Southampton, United Kingdom; ^4^Department of Gastroenterology, Perelman School of Medicine, University of Pennsylvania, Philadelphia, PA, United States

**Keywords:** microcirculation, blood flow, skin, flow-motion, non-linear complexity analysis, NAFLD, liver fibrosis, sympathetic nervous system

## Abstract

**Background/Aims:**

Increasing evidence shows that non-alcoholic fatty liver disease (NAFLD) is associated with dysregulation of microvascular perfusion independently of established cardio-metabolic risk factors. We investigated whether hepatic manifestations of NAFLD such as liver fibrosis and liver fat are associated with microvascular hemodynamics through dysregulation of neurovascular control.

**Methods:**

Microvascular dilator (post-occlusive reactive hyperemia) and sympathetically mediated constrictor (deep inspiratory breath-hold) responses were measured at the forearm and finger, respectively, using laser Doppler fluximetry. Non-linear complexity-based analysis was used to assess the information content and variability of the resting blood flux (BF) signals, attributable to oscillatory flow-motion activity, and over multiple sampling frequencies.

**Results:**

Measurements were made in 189 adults (113 men) with NAFLD, with (*n* = 65) and without (*n* = 124) type 2 diabetes mellitus (T2DM), age = 50.9 ± 11.7 years (mean ± SD). Microvascular dilator and constrictor capacity were both negatively associated with age (*r* = −0.178, *p* = 0.014, and *r* = −0.201, *p* = 0.007, respectively) and enhanced liver fibrosis (ELF) score (*r* = −0.155, *p* = 0.038 and *r* = −0.418, *p* < 0.0001, respectively). There was no association with measures of liver fat, obesity or T2DM. Lempel-Ziv complexity (LZC) and sample entropy (SE) of the BF signal measured at the two skin sites were associated negatively with age (*p* < 0.01 and *p* < 0.001) and positively with ELF score (*p* < 0.05 and *p* < 0.0001). In individuals with an ELF score ≥7.8 the influence of both neurogenic and respiratory flow-motion activity on LZC was up-rated (*p* < 0.0001).

**Conclusion:**

Altered microvascular network functionality occurs in adults with NAFLD suggesting a mechanistic role for dysregulated neurovascular control in individuals at risk of severe liver fibrosis.

## Introduction

Non-alcoholic fatty liver disease (NAFLD) is associated with an increased risk of cardiovascular disease (CVD), of both the macro- and micro-circulations (Lombardi et al., 2019). NAFLD predisposes to increased carotid artery intimal-medial thickness, impaired flow-mediated vasodilation, increased arterial stiffness and coronary artery calcification, independent of multiple cardio-metabolic risk factors such as age, sex and type 2 diabetes mellitus (T2DM) (Oni et al., 2013). NAFLD is also associated with an increased risk of retinal microvascular disease (Liccardo et al., 2015), chronic kidney disease (Byrne and Targher, 2020), and peripheral neuropathy (Williams et al., 2015) in individuals with T2DM, suggesting that the relationship between microvasculopathy and NAFLD may be determined by shared cardio-metabolic risk factors. That said, NAFLD is associated independently with increased prevalence a reduced coronary flow reserve (Vita et al., 2019) and reduced digital microvascular dilator response [reactive hyperemia index (RHI)] (Long et al., 2015; Tuttolomondo et al., 2018). The persistence of the relationship between NAFLD and dysregulation of microvascular hemodynamics beyond established risk factors suggests that hepatic manifestations of NAFLD such as liver fat (Long et al., 2015) and liver fibrosis (Lombardi et al., 2020) may impact directly on microvascular function.

A sustained and variable microvascular perfusion is essential for the optimal delivery of O_2_ and nutrients to match tissue demand (Segal, 2005). Microvascular perfusion is predominately modulated at a local level by rhythmic, oscillatory endothelial (0.0095–0.02 Hz), sympathetic (0.02–0.06 Hz) and myogenic flow-motion activity (0.06–0.15 Hz), additionally influenced by higher frequency respiratory (0.15–0.4 Hz), and cardiac (0.4–1.6 Hz) rhythms (Kvandal et al., 2006). In health, the variability of such non-linear time-varying activity provides the flexibility to cope with changing demands, while disease can involve either a loss or increase of complexity (Vaillancourt and Newell, 2002). Dysregulation of flow-motion activity, measured in non-invasive laser Doppler fluximetry signals, has been observed in a wide range of pathophysiological conditions including obesity (De Jongh et al., 2008; Muris et al., 2014) and hypertension (Gryglewska et al., 2010; Bruning et al., 2015) and insulin resistance (Clough et al., 2011). The variability, or loss of spontaneity of the BF signal, quantified using non-linear approaches (Aboy et al., 2006) has been shown to decline with increasing disease severity both in a primate model of diabetes (Tigno et al., 2011) and in individuals with or at risk of CVD (Liao et al., 2010; Gryglewska et al., 2011). This may also be the case in people with NAFLD (Chipperfield et al., 2019a). However, the mechanistic origins of microvascular perfusion variability (and hence adaptability) and their association with the hepatic manifestations of NAFLD such as liver fibrosis and liver fat has yet to be explored.

Individuals with NAFLD exhibit significant endothelial dysfunction even in the absence of traditional CVD risk factors (Shukla et al., 2017). Additionally, dysregulation of the autonomic nervous system is an important predictor of cardiovascular and metabolic disease risk (Licht et al., 2013), with sympathetic over-activity linked to hypertension (Brook and Julius, 2000), poor glycemic control (Licht et al., 2013), insulin resistance (Svensson et al., 2016), and dyslipidemia (Guarino et al., 2017), all of which may occur in patients with NAFLD. Emerging evidence also suggests a role for the sympathetic nervous system in NAFLD (Hurr et al., 2019). Analysis of heart rate variability (HRV) in individuals with NAFLD indicates an  increased sympathetic tone (Liu et al., 2013). Further, studies in obese mice have shown hepatic steatosis to be associated with robust hepatic sympathetic over-activity; ablation of which reverses the obesity-induced hepatic steatosis (Bruinstroop et al., 2015; Hurr et al., 2019). To what extent dysregulation of sympathetic activity may contribute to an altered flow-motion activity and decline in peripheral microvascular function in people with NAFLD is unclear.

The aim of the study was to investigate microvascular reactivity in the skin of individuals with NAFLD, with and without T2DM, at risk of CVD. To this end we have measured microvascular BF using non-invasive laser Doppler fluximetry (LDF) at two skin sites; that of the well characterized ventral forearm which is under both endothelium-dependent and neurovascular control, and the finger pulp where skin blood flow is largely dominated by arteriovenous anastomoses with dense sympathetic innervation (Braverman, 2000). We have determined the oscillatory activity of the microvascular blood flow signals attributed to local flow-motion using spectral analysis and assessed the variability and irregularity of these oscillatory rhythms using non-linear analysis. We hypothesized that non-linear approaches would provide novel information (Costa et al., 2002; Humeau et al., 2010) on the processes associated with microcirculatory dysfunction in NAFLD and thus whether a declining complexity of the BF signal was associated with dysregulation of local neurovascular control.

## Materials and Methods

### Study Cohort

The manuscript presents data from a secondary analysis of the baseline data from participants who were recruited to two randomized placebo-controlled trials, undertaken in patients with NAFLD. The baseline data only, from participants in both of these trials were combined into a single data-set, in order to undertake the analyses presented in this manuscript. The two trials were the WELCOME (Wessex Evaluation of Fatty Liver and Cardiovascular Markers in NAFLD with OMacor Therapy) (Scorletti et al., 2014) and INSYTE (INvestigation of SYnbiotic TreatmEnt in NAFLD) (Scorletti et al., 2018). All participants had a diagnosis of liver fat on normal clinical grounds with either histological confirmation of NAFLD or imaging evidence of liver fat with exclusion of other liver conditions causing liver fat accumulation. Ethics approvals, informed and written consent were obtained before participants were enrolled into the clinical trials. Data collected from the WELCOME and INSYTE studies included NAFLD severity biomarkers, measures of insulin sensitivity, cardiovascular risk factors, and measures of microvascular function (Scorletti et al., 2014, 2018).

Inclusion criteria for both studies included aged >18 years and having radiological or biopsy-proven NAFLD. Exclusion criteria included decompensated acute or chronic liver disease and viral hepatitis. Participants were also excluded if alcohol consumption was >35 units (1 unit is 7.9 g alcohol) per week for women and >50 units per week for men (Scorletti et al., 2014, 2018). Additional exclusion criteria were pregnancy and breast feeding. Detailed information on different aspects of the studies are available from www.clinicaltrials.gov (WELCOME identifier: NCT00760513, INSYTE identifier: NCT01680640) (Scorletti et al., 2014, 2018).

### Microvascular Blood Flux Signal Capture

Skin microvascular blood flux (BF) signals and skin temperature were recorded simultaneously at the non-dominant forearm using a combined laser Doppler fluximetry LDF^TM^ /temperature probe (VP1T, Moor Instruments Ltd, Axminster, United Kingdom) placed approximately 10 cm proximal to the wrist. A second combined LDF/temperature probe was placed on the pulp of the index finger. The LDF BF signal reflects perfusion in capillaries, arterioles, venules, and dermal vascular plexi (Bollinger et al., 1993). Signals were collected with the participant lying supine with their non-dominant arm resting at heart level as described previously (McCormick et al., 2015). All participants refrained from caffeine containing drinks and food for at least 2 h, and from strenuous exercise for 24 h before testing. Studies were performed in a temperature-controlled room maintained between 22 and 23.5^*o*^C. All participants were acclimatized for at least 30 min prior to testing. Blood pressure was measured prior to signal capture.

Skin BF was recorded continuously (i) at rest for up to 20 min at both the forearm and finger pulp; (ii) during dynamic perturbation of blood flux by three 6 s duration deep inspiratory breath-holds (IBH) to elicit a rapid and transient sympathetically mediated vasoconstriction detected in the cutaneous microvasculature of the finger pulp (Allen et al., 2002; Feger and Braune, 2005); and (iii) during and for 10 min after perturbation of BF by inflation of an automated blood pressure cuff (VMS-PRES, Moor, Axminster, United Kingdom) placed around the upper arm. The cuff was inflated to a supra-systolic pressure of 250 mmHg for 3 min in order to elicit a reactive hyperemia response measured at the ventral surface of the forearm (McCormick et al., 2015).

### BF Signal Analysis

All laser Doppler BF signals were captured at a 40 Hz sampling rate using the manufacturer’s software (moorVMS-PC software, Moor Instruments Ltd, Axminster, United Kingdom).

#### Time-Domain Analysis

BF parameters in the time-domain were determined using moorVMS-PC software (Moor Instruments Ltd, United Kingdom) and expressed in arbitrary perfusion units (PU). These included (i) resting skin blood flux (RF) measured at both the forearm and index finger at baseline over the final 5 min before perturbation; (ii) maximum blood flux at peak hyperemic response to transient ischemia (3 min arterial occlusion) (MF_*arm*_) measured at the forearm (McCormick et al., 2015); and (iii) minimum blood flux achieved during the three x 6 sec breath-holds measured at the index finger (MinF_*f*__*inger*_). The dilator response to reactive hyperemia (RH) at the forearm was expressed as the fold change in BF normalized to baseline ([MF_*arm*_ - RF_*arm*_)]/RF_*arm*_). The sympathetically mediated constrictor response to IBH measured at the finger was expressed as the% change in BF normalized to baseline difference between the percentage change in blood flux normalized to baseline resting finger blood flux ([RF_*finger*_-MinF_*finger*_]/MinF_*finger*_) (L’Esperance et al., 2013). All signal segments were checked to be clear of artifacts prior to analysis.

#### Frequency-Domain Analysis

Prior to spectral analysis, signals were filtered using a finite impulse response low-pass filter with 2 Hz cut-off to attenuate high frequencies beyond the known range of microvascular oscillation. The data segments were then de-trended by removing the mean. Spectral density was estimated by Welch’s method of fast Fourier transform (FFT) with a Hanning window size of 200 s and 50% overlap between windows over continuous 10 min recording periods using MATLAB (R2018a, MathWorks, United Kingdom). The power contribution was evaluated within the frequency range (0.0095–1.6 Hz), divided into frequency intervals corresponding to endothelial (0.0095–0.02 Hz), sympathetic (0.02–0.06 Hz), myogenic (0.06–0.15 Hz), respiratory (0.15–0.4 Hz), and cardiac (0.4–1.6 Hz) activity (Kvandal et al., 2006). Total spectral power was estimated as the sum of absolute power across the five frequency intervals (0.0095–1.6 Hz) and expressed in PU^2^/Hz. Power spectral density (PSD) contribution was calculated relative to total spectral power and is expressed as a fraction between 0 and 1.

#### Non-linear Information and Complexity Analysis

The extent to which the resting BF signals arising from rhythmical flow-motion activity differed from a random sequence was explored using non-linear Lempel-Ziv (LZC) complexity (Lempel and Ziv, 1976) and sample entropy (SE) (Richman and Moorman, 2000; Humeau et al., 2010) analysis to describe the randomness and irregularity of the signal, respectively (Thanaj et al., 2018). Prior to complexity analysis, the original LDF signal was transformed to a binary sequence (Albano et al., 2008). Complexity analysis was applied to 10 min (24,000 samples) of each resting BF signal sampled at 40 Hz. The signals were divided in 15 epochs of length 40 s and the complexity calculated for each epoch (Kuliga et al., 2018; Thanaj et al., 2018). An LZC or SE complexity-index was calculated as the mean of the 15 × 40 s epochs for each sampled signal to provide an index of the dynamic activity modulating the BF signals.

As the BF signal is modulated by at least 5 physiological process, operating at frequencies ranging from 0.001 to 2 Hz, we also measured LZC in multiple time scales in order to account for these multiple, and potentially varying, process scales (Thanaj et al., 2018). LZC was evaluated over multiple sampling frequencies (MLZC) using a coarse-graining approach (Costa et al., 2002; Cerutti et al., 2009). In order to explore how the PSD contribution of each of the frequency bands influenced the information content of the BF signal over these scales, we calculated the Spearman correlations between the power content of each of the five frequency intervals and MLZC over sampling frequencies of 40–1.67 Hz (Chipperfield et al., 2019b).

### Phenotype and Biochemical Measures

Glucose, insulin, total cholesterol, HDL-cholesterol, and triglyceride concentrations were measured in fasting serum or plasma using commercially available kits according to the manufacturers’ instructions. HbA1c was measured by high pressure liquid chromatography (Bio-Rad Laboratories, Irvine, CA, United States). HOMA-IR was calculated from fasting insulin and fasting glucose concentrations. Blood pressure was measured in the non-dominant arm after subjects had become acclimatized and had rested for at least 60 min; the mean of three measurements was calculated. Hypertension was defined by patient history, patient medication for treatment of hypertension, or a blood pressure ≥140/90 mmHg on the average of the three baseline blood pressure measurements at recruitment. Diabetes was diagnosed by patient history, patient treatment for diabetes mellitus or HbA1c measurement ≥48 mmol/mol. Treatments for both conditions were very variable and not consistent between patients because of individual patient needs. Dual-energy X-ray absorptiometry (DEXA), magnetic resonance imaging (MRI) and magnetic resonance spectroscopy (MRS) were undertaken to assess body fat (total body fat, regional body fat, subcutaneous abdominal fat, and visceral fat) and MR spectroscopy to assess liver fat percentage (Scorletti et al., 2018).

The severity of liver fibrosis was estimated using the enhanced liver fibrosis (ELF) score, a commercial blood test using serum concentrations of three fibrosis biomarkers, amino-terminal propeptide of type III procollagen (PIIINP), hyaluronic acid (HA) and tissue inhibitor of matrix metalloproteinase-1 (TIMP-1) (Guha et al., 2008). The ELF score was calculated using the established algorithm i.e., ELF = 2.278 + 0.851 ln (HA) + 0.751 ln (PIIINP) + 0.394 ln (TIMP-1)] (Guha et al., 2008). The ELF test has good performance for diagnosing advanced liver fibrosis and for excluding the presence of advanced liver fibrosis (Byrne et al., 2018; Day et al., 2019). The NAFLD fibrosis score (Angulo et al., 2007) and FIB4 score (Shah et al., 2009) were used to estimate the amount of scarring in the liver [see (Scorletti et al., 2018) for details]. A measure of liver fibrosis detected by transient elastography (FibroScan) (Lombardi et al., 2019) was additionally available in participants from the INSYTE study (*n* = 87) (Scorletti et al., 2018). Overall 10 years risk of CVD was calculated using the Q-RISK 2011 online calculator^1^.

### Statistical Analysis

Statistical analyses were performed using SPSS for Windows version 25.0 (IBM, United States). Data are reported as means and standard deviations for normally distributed variables, or as median and 95%CI for non-normally distributed variables. Pearson and Spearman rank correlation coefficients were used to investigate associations between normally and non-normally distributed variables, respectively. In all cases a value of *p* < 0.05 was taken to indicate significance. Pearson and Spearman rank correlation coefficients were used to investigate associations between normally and non-normally distributed variables, respectively. Spearman’s rho correlations are presented for monotonic non-linear correlation analysis of baseline data. We used a multivariable linear regression model to explore factors that were independently associated with microvascular reactivity and network perfusion complexity. In all cases a value of *p* < 0.05 was taken to indicate significance.

## Results

Blood flux signals were measured in a total of 209 individuals with NAFLD, with and without T2DM, who participated in the WELCOME or INSYTE studies. Excluded from time- and spectral-domain analysis were individuals with T1DM (*n* = 4), microvascular disease (*n* = 1), and those who had participated in both WELCOME and INSYTE studies (*n* = 3). BF signals from a further 12 individuals were found to be unacceptable due to movement artifacts. Of the remaining 189 individuals, 113 (60%) were male and 76 (40%) female. Of these, 65 (34%) had T2DM (mean duration = 5 years, range = 1–30 years) and 80 (42%) were taking antihypertensive medication and 71 (38%) were taking a statin. 23 (12%) of the participants were current smokers. Mean age was 50.9 ± 11.7 years (mean ± SD). The descriptive characteristics of the cohort are summarized in Table 1.

**TABLE 1:** Descriptive characteristics of the combined NAFLD study cohort (*n* = 189).

	NAFLD cohort *n* = 189		
	Mean^#^/Median	SD^#^/95% CI	95% CI
Age (y)	50.9^#^	11.7^#^	
Sex (male/female)	113/76		
T2DM (*n*)	65		
BMI (kg/m^2^)	32.9	31.7	33.8
SBP (mmHg)	135	132	137
DBP (mmHg)	79	77	81
Use of Ca++ channel blockers (n)	17		
H bAlc (mmol/mol)	41	40	43
Fasting plasma glucose (mmol/L)	5.6	5.4	6.0
HOMA-IR	3.5	3.1	4.0
Total body fat (%)	35.8	34.6	37.5
MRS (% liver fat)	24.7	20.7	29.7
Cholesterol (mmol/L)	4.8	4.6	5
LDL (mmol/L)	3.4	3.1	3.8
ELF score	7.91	7.33	8.27
FIB4 score	1.20	1.09	1.25
NAFLD fibrosis score	−1.64	−1.82	−1.18
RF arm (PU)	11.3	10.2	12.3
RF finger (PU)	281	262	307
RH	3.5	3.2	3.8
IBH (%)	62.2	57.3	67.2
LZC-indexarm	0.329	0.318	0.337
SE-index arm	0.137	0.133	0.140
LZC-index finger	0.314	0.297	0.320
SE-index finger	0.137	0.130	0.140

*RH, reactive hyperemic response; IBH, vasoconstrictor response to deep inspiratory breath-hold; LZC, Lempel-Ziv complexity; SE, sample entropy. ^#^Signifies Mean and SD.*

### Univariate Associations Between Microvascular BF Characteristics and Cardiovascular and Metabolic Risk Factors Across the NAFLD Cohort (*n* = 189)

In univariate analysis both microvascular dilator capacity measured at the forearm (RH) and sympathetically mediated constrictor response measured at the finger (IBH) were negatively associated with age (*r* = −0.18, *p* = 0.014 and *r* = −0.20, *p* = 0.007, respectively) as were LZC index and SE index of the resting BF signal measured at both the forearm and finger (all *p* < 0.017) (Table 2). While RH and IBH were negatively associated with ELF score (*r* = −0.16, *p* = 0.038 and *r* = −0.42, *p* < 0.0001, respectively) (Table 2), LZC index and SE index were positively associated with ELF score whether measured at the forearm (LZC: *r* = 0.15, *p* = 0.0367; SE: *r* = 0.29 *p* < 0.0001, respectively) or the finger (LZC: *r* = 0.29 *p* < 0.0001; SE: *r* = 0.29 *p* < 0.0001, respectively) (Table 2). There was no association of any of the microvascular outcomes with measures of cardio-metabolic risk, including sex, obesity, and insulin resistance; nor with liver fat (MRS%) (Table 2). Additionally, microvascular outcomes did not differ significantly between those with and without T2DM.

**TABLE 2:** Univariate Spearman associations between microvascular parameters and cardiovascular risk factors, features of the metabolic syndrome, and markers of NAFLD disease severity in combined cohort (*n* = 189).

Spearman correlations (*n* = 189)		RH arm	IBH finger	LZC-index arm	SE-index arm	LZC-index linger	SE-index finger
Age (year)	*r*	−0.178*	−0.201**	−0.185*	−0.173*	−0.0237**	−0.183*
	*P*	0.014	0.007	0.011	0.017	0.001	0.012
Sex	*r*	0.047	–0.003	0.023	0.063	−0.189**	–0.045
	*P*	0.525	0.964	0.757	0.391	0.010	0.540
BMI (Kg/m^2^)	*r*	–0.076	0.103	0.006	0.083	0.001	0.058
	*P*	0.301	0.171	0.932	0.254	0.987	0.427
Total body fat (%)	*r*	–0.054	0.012	0.054	0.149*	–0.076	0.067
	*P*	0.439	0.873	0.458	0.038	0.293	0.356
MRS (% liver fat)	*r*	–0.039	0.031	0.065	0.069	0.132	0.073
	*P*	0.598	0.681	0.376	0.353	0.072	0.322
NAFLD fibrosis	*r*	–0.119	0.006	–0.141	–0.132	−145*	–0.127
	*P*	0.105	0.937	0.054	0.071	0.048	0.083
ELF fibrosis score	*r*	−0.155*	−0.418**	0.152	0.289**	0.293**	0.292**
	*P*	0.038	0.000	0.037	0.000	0.000	0.000
FIB4 fibrosis score	*r*	–0.138	−0.233**	–0.112	–0.070	–0.021	–0.021
	*P*	0.061	0.002	0.129	0.342	0.776	0.779
T2DM (y/n)	r	0.003	–0.044	–0.026	0.039	–0.082	–0.019
	P	0.934	0.563	0.721	0.598	0.261	0.799
HbA1c (mmol/mol)	*r*	–0.077	−0.226**	–0.024	0.070	0.085	0.147**
	*P*	0.295	0.003	0.741	0.343	0.245	0.045
Fasted glucose (mmol/L)	*r*	0.052	–0.022	–0.047	–0.022	–0.072	–0.036
	*P*	0.479	0.773	0.525	0.764	0.323	0.620
HOMA-IR	*r*	–0.054	0.083	0.084	0.074	–0.006	0.009
	*P*	0.466	0.277	0.260	0.316	0.932	0.904
Use of Ca++ channel blockers	*r*	−0.254**	–0.134	−0.195**	–0.098	–0.028	–0.011
	*P*	0.000	0.075	0.007	0.178	0.698	0.886
RH arm	*r*		0.071	0.0161*	0.088	–0.125	–0.120
	*P*		0.345	0.027	0.228	0.088	0.100
IBH finger (%)	*r*	0.071		–0.052	−0.192*	0–0.227**	0–210**
	*P*	0.345		0.492	0.010	0.002	0.005
LZC-index arm	*r*	0.161*	–0.052		0.821**	0.199**	0.287**
	*P*	0.027	0.492		0.000	0.006	0.000
SE-index arm	*r*	0.088	−0.192*	0.821**		0.382**	0.555**
	*P*	0.228	0.010	0.000		0.000	0.000
LZC-index finger	*r*	–0.125	−0.227**	0.199**	0.382**		0.868**
	*P*	0.088	0.002	0.006	0.000		0.000
SE-index finger	*r*	–0.120	−0.210**	0.287**	0.555**	0.868**	
	*P*	0.100	0.005	0.000	0.000	0.000	

*p < 0.05, **p < 0.005.

We found strong positive associations between ELF score and FIB4 (*r* = 0.47 *p* < 0.0001) and Fibroscan fibrosis score (*r* = 0.29, *p* = 0.007) but not with the Angulo NAFLD fibrosis score (p > 0.05). However, the association between microvascular outcomes and these other also measures of NAFLD severity were weaker compared with those for ELF score (Table 2).

### Multivariable Regression Modeling of Microvascular BF Complexity and Cardiovascular and Metabolic Risk Factors Across the NAFLD Cohort

We examined the relationship between significant univariate predictors presented in the analysis above using multivariable linear regression analysis with LZC index of the resting BF signal measured at the forearm as the outcome variable and age, sex, diabetes status, and ELF liver fibrosis score as independent variables. In this model, LZC-index was significantly associated with age (unstandardized β coefficient = −0.001, 95% CI −0.002, 0.000, *p* = 0.004) and ELF score (unstandardized β coefficient = 0.006, 95% CI 0.000, 0.012, *p* = 0.04). Neither sex (unstandardized β coefficient = 0.001, 95% CI −0.014, 0.016, *p* = 0.892) nor T2DM status (unstandardized β coefficient = −0.001, 95% CI −0.016, 0.013, *p* = 0.872) were independently associated with LZC-index in the model. Addition of the use of Ca^++^ channel blockers (which we have previously shown to influence LZC (Chipperfield et al., 2019a) slightly improved this model.

In a similar model with SE-index as the outcome variable and the same explanatory variables in the model as described above, SE-index was significantly associated with age (unstandardized β coefficient < 0.0001, 95% CI −0.001, 0.000 *p* < 0.0001) and ELF score (unstandardized β coefficient = 0.005, 95% CI 0.002, 0.007 *p* < 0.0001). As with LZC-index, neither sex (unstandardized β coefficient = 0.002, 95% CI −0.004, 0.008, *p* = 0.433) nor T2DM status (unstandardized β coefficient = 0.001, 95% CI −0.005, 0.007, *p* = 0.840) were independently associated with SE-index. Models in which percentage liver fat (MRS%) was substituted for ELF score as an independent variable did not reach significance.

### Impact of Enhanced Liver Fibrosis Score (ELF) on Microvascular Function

As we observed a significant association between ELF score and microvascular outcomes in univariate analysis and in regression modeling, we went on to explore the characteristics of the cohort stratified by an ELF score of <7.8 and ≥7.8, where an ELF score <7.8 has a high probability of excluding patients with advanced liver fibrosis (F3 & F4 fibrosis on liver histology) (Day et al., 2019). Of those with an ELF score <7.8 (*n* = 89) 24 (38%) had T2DM and those in the group with an ELF score ≥7.8 (*n* = 92) 29 (32%) had T2DM (Table 3). The groups did not differ in age or sex, nor in QRISK score [ELF score <7.8: 10.9 (7.1,16.1); ELF score ≥7.8: 9.9 (7.6,14.7) (median (95%CI)].

**TABLE 3:** Characteristics of NAFLD study cohort stratified for ELF score <7.8 (*n* = 89) and ≥7.8 (*n* = 92).

	ELF < 7.8 *n* = 89			ELF ≥ 7.8 = 92			*p*-value ELF < 7.8 vs. ELF ≥ 7.8
	Mean^#^/Median	SD^#^/95% CI		Mean^#^/Median	SD^#^/95% CI		
Age (year mean SD)	50.2^#^	12 5^#^		51.8^#^	10.8^#^		0.3471
Sex (male/female)	58/31			52/40			
T2DM (*n*)	34			29			
BMI (kg/m^2^)	33.7	31.2	34.7	32.4	31.0	33.6	0.6078
SBP (mmHg)	134	128	138	135	132	139	0.1347
DBP (mmHg)	75	72	77	84	81	87	0.0001
Use of Ca++ channel Mockers (n)	8			9			
HbA1 c (mmol/mol)	40.0	37.0	46.0	42.0	40.0	44.0	< 0.0001
Fasting plasma glucose (mmol/L)	6.1	5.5	6.5	5.4	5.2	5.7	0.0043
HOMA-IR	4.0	3.4	4.6	3.2	2.5	3.8	0.0601
Total body fat (%)	34.9	32.0	37.5	36.4	34.6	39.2	0.0428
MRS (% liver fat)	25.0	20.0	34.7	24.0	18.0	32,0	0.5611
ELF score	6.9	6.9	7.0	8.9	8,8	9.1	< 0.0001
FIB4 score	1.01	0.90	1.21	1.26	1.2	1,59	0.0001
NAFLD fibrosis score	−1.17	−1.73	−0.83	−1.8	−2.14	−1.45	0.0981
RF arm (PU)	11.3	9.9	13.2	11.5	1.3	12.7	0.8041
RF finger (PU)	283	248	320	282	258	318	0.6541
RH	3.9	3.4	4.1	3.3	3.0	3.6	0.0600
IBH (%)	72.5	66.2	80.5	48.4	43.1	59.7	< 0.0001
LZC-index arm	0.319	0.306	0.334	0.333	0.320	0.345	0.0130
SE-index arm	0.129	0.121	0.136	0.142	0.136	0.148	0.0001
LZC-index finger	0.294	0.274	0.31	0.330	0.319	0.342	< 0.0001
SE-index finger	0.127	0.12	0.133	0.140	0.134	0.142	0.0001

*RH, reactive hyperemic response; IBH, vasoconstrictor response to deep inspiratory breath-hold; LZC, Lempel-Ziv complexity; SE, sample entropy. ^#^ signifies Mean and SD.*

#### Microvascular Reactivity

**FIGURE 1:**
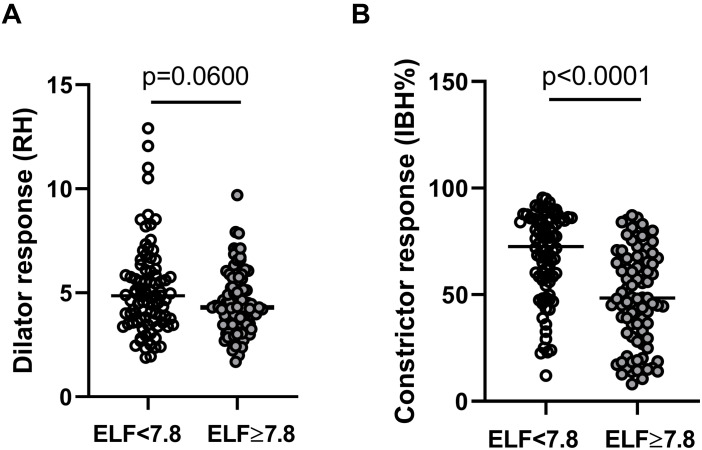
Skin microvascular **(A)** dilator response to reactive hyperemia (RH) measured at the forearm and **(B)** constrictor response to deep inspiratory breath-hold (IBH%) measured at the finger in individuals with NAFLD with an ELF score of <7.8 (open circles *n* = 89) and ≥7.8 (filled circles *n* = 92).

There was no significant difference in resting microvascular blood flux (RF_*arm*_ and RF_*finger*_) or microvascular conductance (MVC = BF/mean arterial BP) between the two groups. Maximal MVC at peak hyperemic BF was significantly lower in those with an ELF score >7.8 (ELF score <7.8: 0.59 (0.52,0.67) PU/mmHg; ELF score ≥7.8, 0.49 (0.43,0.57) PU/mmHg, *p* = 0.0022). There was also a trend toward a difference in RH response between those with an ELF score of <7.8 and ≥7.8 (∼12%) (*p* = 0.06) (Table 3). The constrictor response to breath-hold (IBH) measured at the finger was significantly smaller in individuals at a greater risk of significant liver fibrosis (ELF > 7.8), falling by more than 33% (*p* < 0.0001) (Table 3 and Figure 1).

#### Spectral Power of Resting BF Signals

**FIGURE 2:**
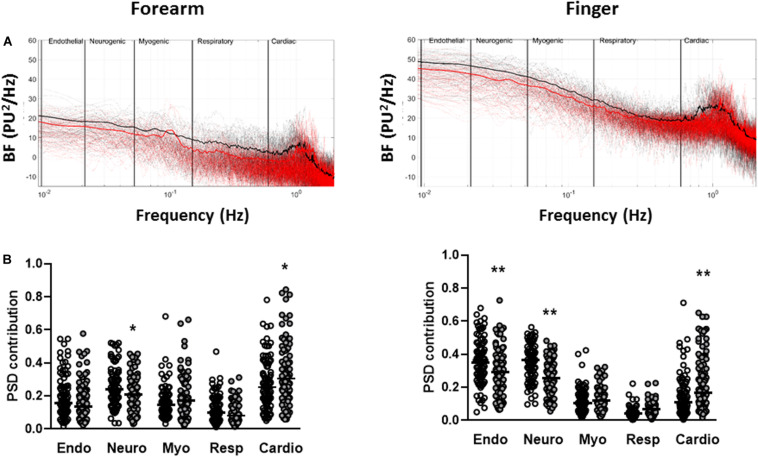
Spectral-domain analysis of resting blood flux signals measured at the forearm and finger. **(A)** Individual (dotted) and mean (solid) spectra of spectral density across the five frequency bands corresponding to endothelial (0.0095–0.02 Hz), sympathetic (0.02–0.06 Hz), myogenic (0.06–0.15 Hz), respiratory (0.15–0.4 Hz), and cardiac (0.4–1.6 Hz) activity in individuals with ELF score <7.8 (black *n* = 89) and ELF score ≥7.8 (red *n* = 92). **(B)** Relative power spectral density (PSD) contribution of each of the spectral bands to total power for ELF score <7.8 (open circles *n* = 89) and ELF score ≥7.8 (filled circles *n* = 92). Bar = median. *p < 0.05, **p < 0.01.

The total spectral power of the resting BF signals was positively associated with resting BF (forearm, *r* = 0.63 *p* < 0.0001; finger *r* = 0.39 *p* < 0.0001) and negatively associated ELF score measured at both the forearm (*r* = −0.18, *p* = 0.017, *n* = 181) and the finger (*r* = −0.31 *p* < 0.0001, *n* = 181). Total spectral power was negatively associated with age at the finger (*r* = −0.16, *p* = 0.031) but not the forearm. There was no association between measures of dyslipidemia or dysglycemia and total spectral power at either skin site (all *p* > 0.05). Power spectra of the BF signals from individuals dichotomized by ELF score exhibited multiple oscillatory components at both skin sites (Figure 2A). While individual spectra showed no obvious patterns associated within each group, the mean spectra for each group showed some discernible peaks within each frequency band, most notably in the cardiac band around 1 Hz, consistent with a resting heart rate of ∼60 beats per minute. In univariate analysis increasing ELF score was associated negatively with the relative PSD contribution of the endothelial (*p* < 0.04), neurogenic (*p* < 0.03) and respiratory (*p* < 0.05) frequency bands and positively with that of the cardiogenic frequency band (*p* < 0.005) at both skin sites. There was no significant association between ELF score and myogenic PSD contribution at either skin site (*p* > 0.05). There was however, a strong association between PSD contribution and age at both sites (all *p* < 027). The relative PSD contributions of the five frequency bands to total spectral power measured at the two skin sites and stratified by ELF score are shown in Figure 2B.

The sympathetically mediated IBH response was strongly and positively associated with the relative PSD contribution of the neurogenic band both measured at the finger (*r* = 0.42 *p* < 0.0001, *n* = 181). There was no significant association between RH and the relative PSD contribution in any of the low frequency spectral bands both of which were measured at the forearm. The use of calcium channel blockers was positively associated with PSD contribution in the cardiac band at both the forearm (*r* = 0.34 *p* < 0.0001) and the finger (*r* = 0.16, *p* = 0.028).

#### Information Content and Complexity of Resting BF Signals

**FIGURE 3:**
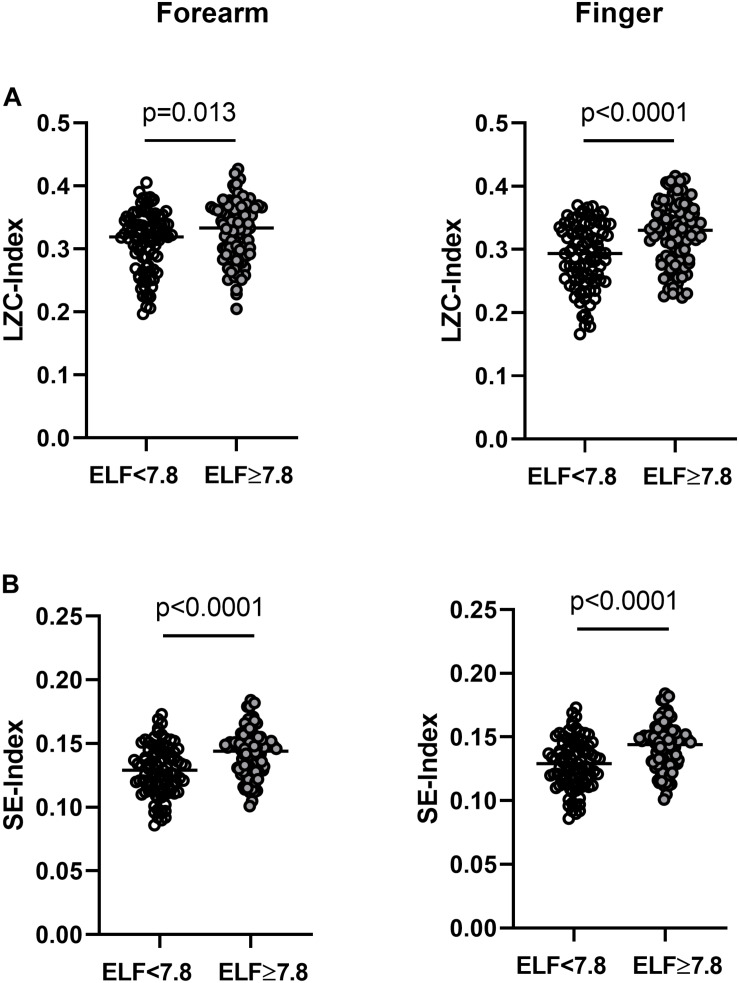
**(A)** Lempel-Ziv complexity index (LZC-index) and **(B)** Sample entropy index (SE-index) of the blood flux signals measured at the forearm and finger in individuals with NAFLD with an ELF score <7.8 (open circles *n* = 89) and ≥7.8 (closed circles *n* = 92). Horizontal bar = median.

The LZC and SE of the resting BF signals measured at the forearm and finger were relatively constant over the 15 × 40 s epochs in all individuals (data not shown). At both skin sites, the BF signals appeared more variable (i.e., had a higher LZC- and SE-index) in those at greater risk of severe liver fibrosis (Table 3 and Figure 3). There was a positive relationship between the LZC-index of the BF signal and the RH response measured at the forearm (LZC, *r* = 0.16, *p* = 0.027) and a negative association between both LZC-index and SE-index and IBH, all measured at the finger (LZC, *r* = −0.21, *p* = 0.005; SE *r* = −0.23, *p* = 0.002) (Table 2).

LZC-index measured at the forearm was positively associated with the relative power of the endothelial (*p* < 0.0001), neurogenic (*p* = 0.019) and respiratory (*p* < 0.0001) frequency bands and negatively with the cardiogenic band (*p* < 0.0001). LZC-index measured at the finger was positively associated with the relative power of the myogenic (*p* < 0.0001) and respiratory (*p* < 0.0001) bands and negatively associated with the relative power of the endothelial band (*p* = 0.045).

**FIGURE 4:**
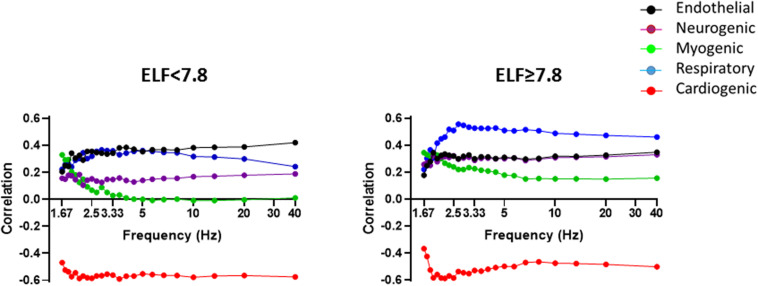
Spearman correlations between BF signal LZ complexity and PSD contribution of the five frequency bands corresponding to endothelial (0.0095–0.02 Hz) (black), sympathetic (0.02–0.06 Hz) purple), myogenic (0.06–0.15 Hz) (green), respiratory (0.15–0.4 Hz) (blue), and cardiac (0.4–1.6 Hz) (red) activity with increasing sampling frequency, measured in the resting forearm BF signal in individuals with an ELF score <7.8 (*n* = 89) and ≥7.8 (*n* = 92).

To understand how the spectral components of the BF signal influence its information content and hence complexity, we examined the correlations between the power bands of the BF signal and MLZC (Figure 4). As shown previously the influence of all five frequency bands remained relatively constant once the Nyquist frequency of the original BF signal (3.2 Hz) was reached or passed (Chipperfield et al., 2019b). There were marked between-group differences in the influence of the individual spectral bands on BF signal complexity. At the forearm, endothelial, neurogenic, myogenic and respiratory band activity contributed positively, and the relatively regular cardiac band activity negatively, to the information content of the BF signal (Figure 4). In individuals with an ELF score ≥7.8 the influence of the endothelial band on signal complexity was down-rated (*p* = 0.0003), whereas the influence on LZC of the neurogenic (*p* < 0.0001), myogenic (*p* < 0.0001) and respiratory (*p* < 0.0001) bands was up-rated. The negative modulation of the information content of the BF signal by the cardiac band was reduced in those with an ELF score ≥7.8 (*p* = 0.0014) but remained significant in both groups. Similar trends were seen at the finger with the positive influence on LZC of the neurogenic and myogenic bands up-rated (*p* < 0.0001) in those with an ELF score ≥7.8.

## Discussion

Our novel results show that ELF score (a measure of liver fibrosis) was independently associated with microvascular functionality and network flexibility in individuals with NAFLD both with and without T2DM, who are at risk of CVD but are without overt microvascular disease. Importantly, while 34% of the NAFLD cohort had T2DM, our finding of an altered microvascular network functionality and flexibility was independent of the presence of T2DM. Microvascular reactivity was not associated with measures of hepatic steatosis or body fat. Individuals with higher ELF scores (in keeping with increased risk of liver fibrosis) exhibited greater BF signal complexity, attributable in part to an uprating of the influence of the frequency modulators associated with local and systemic neurovascular control of flow-motion. Taken together these data are strongly indicative of a dysregulated sympathetic activity in NAFLD that contributes to microvascular dysfunction.

We assessed skin microvascular function in 189 participants in baseline data from two randomized placebo-controlled trials (Scorletti et al., 2014, 2018). Each participant had biopsy-proven or imaging-confirmed NAFLD and other causes of chronic liver disease were excluded in all participants. 34% of them had T2DM of duration ranging from 1 to 30 years. No participants had overt microvascular pathology (neuropathy, nephropathy, or retinopathy). Both cutaneous microvascular dilator and constrictor capacity in this cohort were negatively associated with liver fibrosis, most notably with the ELF test score which has good performance for diagnosing advanced liver fibrosis and for excluding the presence of advanced liver fibrosis (Byrne et al., 2018; Day et al., 2019). We stratified our cohort by an ELF score <7.8 because it has been shown that below the threshold there is a high probability that subjects do not have advanced liver fibrosis (Byrne et al., 2018).

The associations between microvascular functionality and ELF score were independent of cardiovascular risk factors, including age, T2DM, and body fat. They were also independent of liver steatosis. Importantly, and of relevance to the negative association that we report here, is the observation by Yilmaz et al. (2010) that liver fibrosis was an independent predictor of impaired coronary microvascular flow reserve. Yilmaz et al. (2010) further showed that this relationship between coronary microvascular dilator capacity and the histological NASH fibrosis score persisted even after taking age, sex, diabetes, the metabolic syndrome, and other potential confounders into consideration. The observed decline in cutaneous microvascular function in NAFLD is consistent with that measured using peripheral arterial tonometry (PAT) (Tuttolomondo et al., 2018), a relationship that also persisted after adjustment for body mass index and visceral adipose tissue (Long et al., 2015).

We observed a negative association between the sympathetically mediated cutaneous vasoconstrictor response to deep inspiratory breath-hold and ELF fibrosis score in people with NAFLD but without diabetic neuropathy (Quattrini et al., 2007). The decline in vasoconstrictor response in NAFLD is consistent with that observed in obese individuals and in those with non-insulin-dependent diabetes, assessed by deep inspiratory breath-hold or by the sitting-to-standing test (Valensi et al., 1997, 2000). However, the negative association between IBH and ELF fibrosis score in NAFLD was independent of these potential confounders and also of liver steatosis. Previous studies in obese mice have shown both dyslipidemia (Hurr et al., 2019) and hepatic steatosis (Bruinstroop et al., 2015; Hurr et al., 2019) to be positively associated with hepatic sympathetic over-activity and enhanced hepatic vasoconstrictor hyper-reactivity (Van der Graaff et al., 2018). Our finding in people with NAFLD of a negative association between the sympathetically mediated IBH response in the skin microvasculature and liver fibrosis scores that was independent of liver fat appears contrary to these findings in mice. The skin is an important thermoregulatory organ and glabrous skin such as that on the palms and fingertips has been shown to demonstrate a degree of dynamic autoregulation (Wilson et al., 2005). It is thus possible that those at lower risk of liver fibrosis exhibited an adaptive modulation of sympatho-vagal activity to preserve resting BF and the vasoconstrictor response without excessive autonomic stress (Passino et al., 1996). Dysregulation of sympathetic activity with increasing disease severity may serve to limit, rather than enhance, the peripheral microvascular constrictor response to IBH.

A decline in microvascular functionality is widely associated with variations in the amplitude and relative contribution of the low frequency oscillations, with flow patterns differing according to the time course and severity of disease (Rossi et al., 2006, 2008; Muris et al., 2014). However, there is a lack of consensus on direction of change in oscillatory components of the BF signal and the balance between the absolute or relative power in the frequency bands with much appearing cohort specific and measurement site dependent (Clough et al., 2017). In the current study we observed an overall decrease in total spectral power of the BF signals with increasing risk of severe liver fibrosis, which together with the negative association between ELF score and the PSD contribution of the local endothelial and neurogenic and the respiratory frequency bands, is consistent with a mechanistic decline in microcirculatory function with increasing disease severity.

There was no significant association between ELF score and myogenic PSD contribution, indicative of vasomotion (Aalkjaer and Nilsson, 2005), at either skin site suggesting that there was no decline in vascular smooth muscle activity with increasing risk of liver fibrosis in our cohort. The reason for the observed positive association between PSD contribution of the cardiogenic frequency band and ELF score is less clear. It is most probable that this is related to an increase in the visibility of this frequency band with the decline in other flow-motion activities. Alternatively, but as yet unproven, it is possible that in individuals at greater risk of severe liver fibrosis, transmission of the cardiac rhythm within the microvascular network may be enhanced by differences in systolic or diastolic blood pressure, large vessel stiffness and/or small vessel rarefaction.

Non-linear complexity analysis provides a measure of the information content and hence variability and regularity of a signal, and both LZC- and SE- complexity indices have been used as indicators of functional (dys)regulation of the microvasculature in individuals at increasing risk of cardio-metabolic disease (Liao et al., 2010; Figueiras et al., 2011; Tigno et al., 2011; Chipperfield et al., 2019a). In the current study, LZC and SE complexity of the BF signals measured at the forearm and the finger, increased with increasing NAFLD severity i.e., microvascular perfusion became more random and less regular. The increase in randomness and irregularity was associated with a decline in function. Previous studies in at-risk groups, in whom microvascular impairment has been demonstrated using reactivity tests, have shown LZC and SE of BF signals either to increase (Figueiras et al., 2011) or to decrease (Humeau et al., 2008; Liao et al., 2010; Figueiras et al., 2011; Tigno et al., 2011; Chipperfield et al., 2019a). This suggests that microvascular dysfunction can involve either a loss or an increase in complexity (Vaillancourt and Newell, 2002). Interestingly, Chipperfield et al. (2019a) in their validation study of complexity-based methods as discriminators of increasing CVD risk, found little difference in LZC-index of BF signal measured at the forearm in 50 individuals with NAFLD, grouped for the absence or presence of T2DM. This observation is not inconsistent with the current finding that the associations between LZC- and SE-index and ELF score were independent of T2DM, so lending further support for a direct influence of the hepatic manifestations of NALFD on microvascular perfusion.

Examination of the information content of the BF signals revealed clear and significant differences in LZC in individuals with an ELF score <7.8 and ≥7.8 that became more pronounced at certain sampling frequencies (Figure 4). These differences are indicative of an uprating in the influence of the low frequency neurogenic and myogenic power and higher frequency respiratory power on the information content of the BF signals from those at greater risk of severe liver fibrosis. This uprating in the influence of these frequency bands on signal complexity appears inconsistent with the negative association between the relative PSD contribution of these frequency bands and ELF score. However, skin sympathetic nerve activity is known to be modulated by respiration, and cutaneous vasoconstrictor neurones to be temporarily coupled to both respiratory and cardiac oscillations (Fatouleh and Macefield, 2013). An altered central respiratory coupling is also known to contribute to the maintenance of elevated sympathetic vasomotor activity in disease (Simms et al., 2010). Thus, the increase in randomness and loss of regularity of flow-motion activity together with the decline in functionality appear consistent with a shift in (or loss of) the system “set-point,” consequent to an altered sympatho-vagal activity in NAFLD. Further studies are needed to test this hypothesis.

Finally, we observed a down-rating in the influence of the cardiac power on the information content of the BF signal as risk of disease severity increased, although it remained significant in both groups. A decline in the influence of the more periodic, relatively regular heartbeat should result in an increase in signal complexity. HRV is known to contribute to complexity of the BF signal (Sassi et al., 2015; Wang et al., 2018) and HRV has been shown to be increased in obese individuals in whom there is a relative predominance of sympathetic activity in both the time and frequency domains (Rossi et al., 2015). HRV has also been shown to be increased in individuals with NAFLD (Kumar et al., 2016) independently of conventional cardiovascular risk factors and insulin resistance (Liu et al., 2013). Thus, it is possible that the down-rating of the association between cardiac power contribution and BF signal complexity was in part due to an increase in HRV. Unfortunately, we have no synchronous measure of HRV and LD BF in our cohort. Further, 17 of the study participants were taking calcium channel blockers and inclusion of their use improved our multiple regression models of BF signal complexity in NAFLD. The negative association between LZC-index measured at the forearm and calcium channel blocker use is consistent with that reported previously by Chipperfield et al. (2019a) and supports the negative influence of the periodic heartbeat on BF signal complexity through their action of peripheral myogenic tone.

### Study Strengths and Limitations

There are several strengths of our study, not least that we have characterized in depth 189 individuals with NAFLD. We found good coherence between ELF score and other measures of liver fibrosis in our cohort, including FIB4 and Fibroscan fibrosis score which, like the ELF score, were negatively associated with our measures of microvascular function. Microvascular BF and reactivity were assessed at two skin sites and signals characterized using well validated time, frequency and non-linear analysis approaches. The application of non-linear analysis to signals derived from blood flow through the microvascular networks has provided a novel mechanistic insight into the effects of the hepatic manifestations of NAFLD on microvascular function. A limitation of our study is that we have not been able to confirm the presence of liver fibrosis in this cohort with liver histology. However, that said, we chose a threshold of ELF score below which the score has proven excellent diagnostic performance for excluding the presence of advanced liver fibrosis. The data used in this study were from a secondary analysis of the baseline data from participants who were recruited to two randomized placebo-controlled trials, undertaken in patients with NAFLD. The study therefore lacked a “control” group without NAFLD. As evidenced in Figure 2, the power-frequency profiles do not show a dominant frequency of oscillation in the defined low frequency bands, in contrast to the clear peak around the 1 Hz cardiac band. It is thus difficult to explore the impact of NAFLD on flow-motion activity through the relative PSD contributions. Here, we have used the FFT to estimate the PSD contributions of the frequency bands. It should be noted that as the LDF signal is non-stationary, particular care is required when parameterizing Welch’s method to achieve reliable spectral estimates. Wavelet-based approaches provide time-frequency localization which may be advantageous, particularly when examining the lower frequency oscillations. The range and borders of the frequency bands that we have used in our spectral analysis of the resting BF signals were defined previously by others (Kvandal et al., 2006), and it is possible that the spectral profiles comprise multiple frequency components that may vary, for example, with age or pathological state (Grinevich et al., 2019). In our NAFLD cohort, both microvascular reactivity and spectral power relating to flow-motion activity were strongly and negatively associated with age, a well-recognized risk factor for microvascular dysfunction (Payne and Bearden, 2006), including in the liver (Warren et al., 2008).

## Conclusion

The changes in the dynamic characteristics of the BF signal are indicative of loss of system flexibility in NAFLD that may serve to constrain functionality and give rise to a mismatch between perfusion and demand, contributing to disease risk and/or severity. The decline in system flexibility that was attributable in part to dysregulated neurovascular control, persisted beyond established cardio-metabolic risk factors (e.g., T2DM) and was independently associated with a validated test for the presence of liver fibrosis. These data highlight the importance of an altered sympatho-vagal activity in NAFLD and we suggest that indices of signal complexity derived from the vasculature may offer a method for risk stratification of autonomic dysfunction.

## Data Availability Statement

The datasets generated for this study are available on request to the corresponding author.

## Ethics Statement

The studies involving human participants were reviewed and approved by Southampton and South West Hampshire Local Research Ethics Committee (REC: 08/H0502/165 and REC: 12/SC/0614). Registered with www.clinicaltrials.gov (WELCOME identifier: NCT00760513, INSYTE identifier: NCT01680640). The patients/participants provided their written informed consent to participate in this study.

## Author Contributions

GC, AC, ES, PC, and CB were involved in the conception and design of the study. GC and ES collected the data. GC, AC, MT, ES, and CB analyzed the data. GC, AC, MT, and CB interpreted the results. GC and AC prepared the first draft of the manuscript. All authors were involved in the revision of the draft manuscript and have approved the final content. All persons designated as authors qualify for authorship, and all those who qualify are listed.

## Conflict of Interest

The authors declare that the research was conducted in the absence of any commercial or financial relationships that could be construed as a potential conflict of interest.

## Footnotes

1 www.qrisk.org

## Abbreviations

BF: blood flux
CVD: cardiovascular disease
ELF: enhanced liver fibrosis
FFT: fast Fourier transform
HF: high frequency
HRV: heart rate variability
IBH: inspiratory breath-hold
LDF: laser Doppler fluximetry
LF: low frequency
LZC: Lempel and Ziv complexity
MLZC: multiscale Lempel and Ziv complexity
MSE: multiscale sample entropy
NAFLD: non-alcoholic fatty liver disease
PSD: power spectral density
PU: perfusion units
RF: resting flux
RH: post occlusive reactive hyperemia
SE: sample entropy
T2DM: type 2 diabetes mellitus.
